# Natural Products to Counteract the Epidemic of Cardiovascular and Metabolic Disorders

**DOI:** 10.3390/molecules21060807

**Published:** 2016-06-22

**Authors:** Birgit Waltenberger, Andrei Mocan, Karel Šmejkal, Elke H. Heiss, Atanas G. Atanasov

**Affiliations:** 1 Institute of Pharmacy/Pharmacognosy and Center for Molecular Biosciences Innsbruck (CMBI), University of Innsbruck, 6020 Innsbruck, Austria; birgit.waltenberger@uibk.ac.at; 2 Department of Pharmaceutical Botany, Iuliu Hațieganu University of Medicine and Pharmacy, 400012 Cluj-Napoca, Romania; mocan.andrei@umfcluj.ro; 3 Department of Natural Drugs, Faculty of Pharmacy, University of Veterinary and Pharmaceutical Sciences Brno, 612 42 Brno, Czech Republic; karel.mejkal@post.cz; 4 Department of Pharmacognosy, University of Vienna, 1090 Vienna, Austria; elke.heiss@univie.ac.at; 5 Institute of Genetics and Animal Breeding of the Polish Academy of Sciences, 05-552 Jastrzebiec, Poland

**Keywords:** natural products, cardiovascular disease, metabolic disorders, diabetes mellitus, statins, biguanides, dietary constituents, coffee, molecular targets

## Abstract

Natural products have always been exploited to promote health and served as a valuable source for the discovery of new drugs. In this review, the great potential of natural compounds and medicinal plants for the treatment or prevention of cardiovascular and metabolic disorders, global health problems with rising prevalence, is addressed. Special emphasis is laid on natural products for which efficacy and safety have already been proven and which are in clinical trials, as well as on plants used in traditional medicine. Potential benefits from certain dietary habits and dietary constituents, as well as common molecular targets of natural products, are also briefly discussed. A glimpse at the history of statins and biguanides, two prominent representatives of natural products (or their derivatives) in the fight against metabolic disease, is also included. The present review aims to serve as an “opening” of this special issue of *Molecules*, presenting key historical developments, recent advances, and future perspectives outlining the potential of natural products for prevention or therapy of cardiovascular and metabolic disease.

## 1. Introduction

It is well known that natural products have been a valuable source of therapeutic agents for millenia and even today, many medicines are natural products or their derivatives [1]. Although natural products have played an important role in lead discovery [1], nowadays the pharmaceutical industry tends to not prioritize natural product research anymore [2]. Instead, common strategies in industry are high throughput screening (HTS) of synthetic compound databases and structural modifications of existing leads. However, the HTS and combinatorial chemistry approaches followed by many pharmaceutical companies have not been very successful. Furthermore, even stakeholders in industry still see a high potential in natural products as drug leads [3]. In line with this view, the number of scientific studies in the area of natural products research is increasing rapidly [1]. The 2015 Nobel Prize in Physiology or Medicine, which was awarded to Youyou Tu, William C. Campbell, and Satoshi Ōmura for the discovery of natural products for the treatment of tropical parasitic diseases [4,5], might be considered emblematic for the revival of natural product drug discovery. It clearly shows the therapeutic value of natural products and underlines that natural products are an effective source of new drugs.

This review is intended to serve as an “opening” for the *Molecules* special issue entitled “Effects of Natural Products in the Context of Cardiometabolic Disease”. It presents selected prominent illustrative examples of natural products with effects on cardiovascular and metabolic disorders, and is far from being comprehensive. A focus is set on medicinal plants and terrestrial plant-derived natural products, and readers are referred to other recent reviews for an overview on natural products with relevant activities from seaweeds and other marine organisms [6,7,8,9].

## 2. Cardiovascular and Metabolic Disorders—A Global Health Problem

The metabolic syndrome is considered to be a progressive pathophysiological state which is clinically manifested by a cluster of interrelated risk factors (abdominal obesity, atherogenic dyslipidemia, increased blood pressure, insulin resistance, pro-inflammatory and pro-thrombotic state) and associated with an increased expectation for developing diabetes mellitus type 2 and atherosclerotic cardiovascular disease [10,11]. Atherosclerosis, alongside with hypertension, is the main cause of cardiovascular disease representing the leading cause of death in the world. A sedentary life-style together with a diet comprising high calorie intake in westernized societies render the disease prevalence high and atherosclerosis is therefore the underlying cause of approximately 50% of all deaths [12]. Moreover, the prevalence of cardiovascular disease in the world is rising globally and according to the World Health Organization (WHO), this increasing tendency is likely to continue in the next years. While in 2012, cardiovascular disease caused 17.5 million deaths, it is projected to be responsible for 22.2 million deaths in 2030 [13].

Diabetes mellitus is considered one of the most common chronic metabolic diseases in nearly all countries. Especially the prevalence of diabetes mellitus type 2, which accounts for around 90% of all diabetes cases worldwide, continues to increase due to the changing lifestyles that involve reduced physical activity and increased incidence of obesity. In 2014, the prevalence of diabetes reported by the WHO was estimated to be 9% among adults aged 18+ years while in 2012, an estimated 1.5 million deaths were directly caused by this disease [14]. According to projections, its prevalence will further increase [15], becoming the 7th leading cause of death by 2030 [16].

## 3. Increasing Scientific Interest in Natural Products with Potential Application in Cardiovascular and Metabolic Disorders

Considering the huge morbidity and mortality burden related to cardiometabolic disorders with no end in sight, there is a high interest in the discovery of novel compounds as well as novel pharmacological targets that might be effective in the treatment or prevention of cardiovascular and/or metabolic disorders. Although natural product drug discovery often requires more effort compared to HTS and combinatorial chemistry, nature is still considered as the most productive source of potential drug leads for new medicines [3].

**Figure 1:**
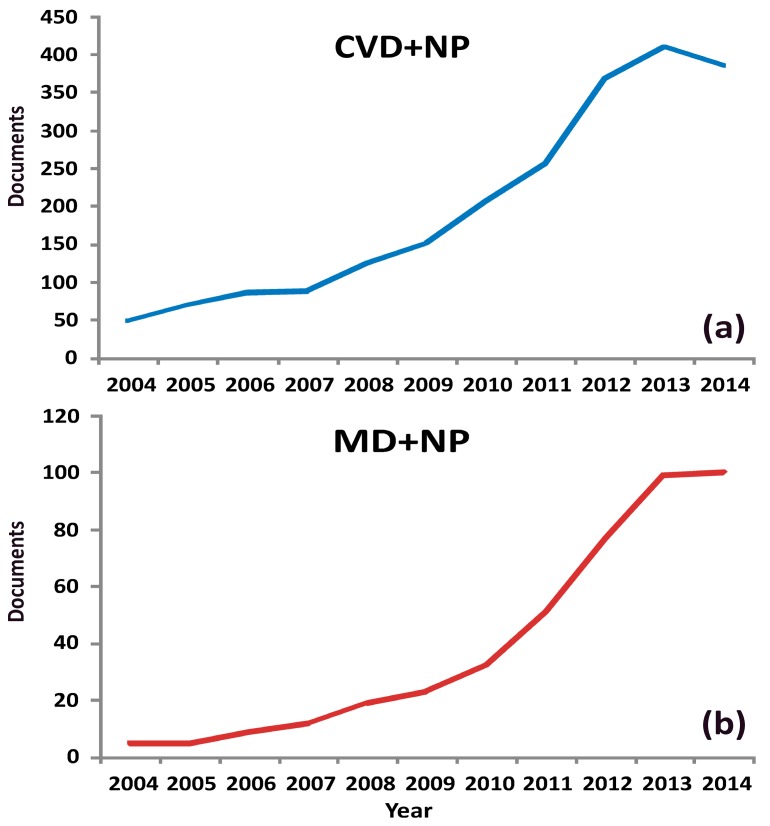
Annual number of publications resulting from the search with the keywords “cardiovascular disease” and “natural products” (CVD + NP) (**a**) and “metabolic disease” and “natural products” (MD + NP) (**b**), (Scopus, January 2016).

In recent decades, herbal remedies and natural products have undisputedly attracted much research attention in the context of prevention or treatment of cardiovascular and metabolic disease [17,18,19]. Thus, when searching Scopus using the keywords “cardiovascular disease” and “natural products” (CVD+NP) or “metabolic disease” and “natural products” (MD+NP) it becomes evident that the scientific interest in these areas increased exponentially in the period 2004–2014 (Figure 1).

## 4. Plants Traditionally Used in the Context of Cardiovascular and Metabolic Disorders

Millenary civilizations rely on plants or other natural resources to sustain or restore health, and in various situations they still represent interesting therapeutic alternatives to synthetic drugs. According to the WHO, over 100 million Europeans and many more people in Africa, Asia, Australia, and North America are users of traditional and complementary medicine. Especially in Africa and some developing countries, traditional medicine is often the primary source of health care [20].

Along with herbal extracts and natural products with validated efficacy and safety proven by randomized controlled clinical trials (further discussed in chapter 5), many other medicinal plants are used world-wide to alleviate cardiovascular and metabolic complaints. Table 1 provides an overview of selected traditionally used plants and their targeted indications.

There is no doubt that medicinal plants and natural products are used for the treatment or prevention of cardiovascular and metabolic disorders, also with rising popularity in western societies. However, in most cases the expected health benefits are not scientifically proven by rigorous clinical trials. Hence, it is vital to provide robust scientific evidence for clinical efficacy and safety.

## 5. Herbal Products in Recruiting Clinical Trials Targeting Indications Related to Cardiovascular and Metabolic Disorders

Many medicinal plants and natural products are considered by the public as a safe, natural, and cost-effective alternative to synthetic drugs without unambiguous proof by randomized controlled clinical trials. On this background, there is an increased interest in the development of products with validated efficacy and safety, similar to the recently FDA-approved botanical drugs Veregen^®^ (sinecatechins; green tea (*Camellia sinensis* (L.) Kuntze) leaf extract), Fulyzaq^®^ (crofelemer; extract from the red latex of the Dragon’s blood tree (*Croton lechleri* Müll.Arg.)), and Grastek^®^ (Timothy grass (*Phleum pretense* L.) pollen allergen extract) [177,178]. Some herbal extracts and pure compounds are currently undergoing clinical trials for cardiometabolic indications; an overview is presented in Table 2.

## 6. Dietary Constituents with Potential Benefits in the Context of Cardiovascular and Metabolic Disorders

Ample evidence demonstrates that dietary patterns can affect the development of cardiovascular and metabolic disorders [179,180]. The reduced intake of highly processed foods by replacing them with fruits, nuts, seeds, vegetables, and legumes [181] is considered health promoting. The latter dietary constituents are free of food additives, low in salt content, and rich in phenolics, carotenoids, fibers, minerals, and unsaturated fats. They possess antioxidant effects, lower glycemic indices, and normalize levels of cholesterol in blood. The traditional Mediterranean diet is one example, which is associated with longer life expectancy, lower rates of cardiovascular and metabolic disorders, and even lower rates of certain cancers [182]. This diet is characterized by an abundance of seasonally fresh plant foods (fruits, vegetables, beans, nuts, seeds, *etc.*), minimal food processing, olive oil, and wine consumed in low to moderate amounts, normally with meals [19,182,183].

**Figure 2:**
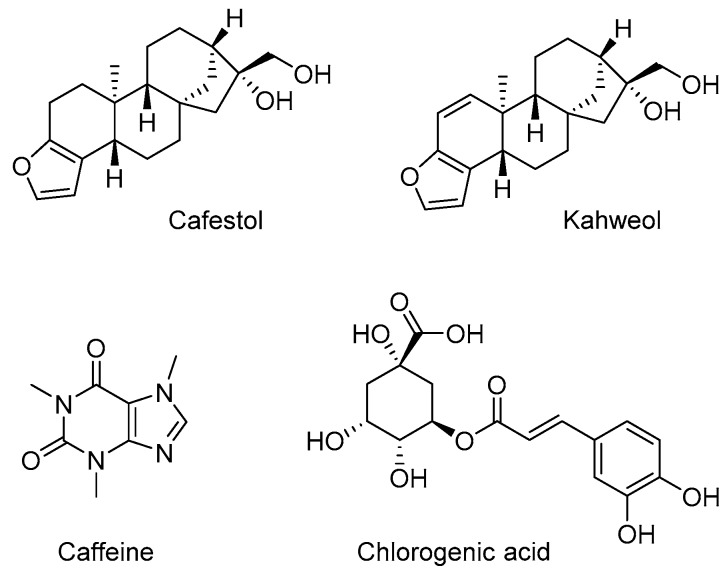
Chemical structures of bioactive compounds found in coffee.

Another example for a presumably health promoting dietary constituent is coffee, one of the most popular beverages worldwide. It exhibits a range of bioactivities and potential health benefits. Since coffee drinking is very common in Western societies, its bioactivities and in particular its impact on cardiovascular and metabolic parameters have been widely investigated [184,185,186,187,188,189,190]. Compared to non-drinkers, coffee consumption of one to five cups/day was associated with lower risk of mortality, while coffee consumption of more than five cups/day did not affect mortality risk. Additionally, coffee consumption (with or without caffeine) was associated with significantly lower death risk due to cardiovascular disease, neurological disorders, and suicide [191]. It was also linked to a lower risk of diabetes mellitus type 2, independent of race, geographic distribution and gender of the studied populations [192]. Major bioactive ingredients in coffee include phenolics (chlorogenic acid and its isomers), diterpenes (cafestol and kahweol), and caffeine (Figure 2). Coffee is considered to be a very prominent source of phenolic compounds, and it appears that it is the number one source of dietary antioxidants in the US [193,194]. The total phenolic content per cup of coffee ranges between 200 and 550 mg, with chlorogenic acid being the main phenolic compound [192].

Chlorogenic acid intake leads to lower blood glucose and insulin concentrations 15 minutes after ingestion [195]. In streptozocin-nicotinamide induced diabetic rats, a dose of 5 mg chlorogenic acid/kg body weight exerts antidiabetic effects [195,196]. Additionally, coffee phenolics can intensify energy metabolism and decrease lipogenesis by down-regulation of SREBP-1c and related molecules [197]. Moreover, coffee phenolics are able to modulate whole-body substrate oxidation by suppressing postprandial hyperglycemia and hyperinsulinemia [198].

**Figure 3:**
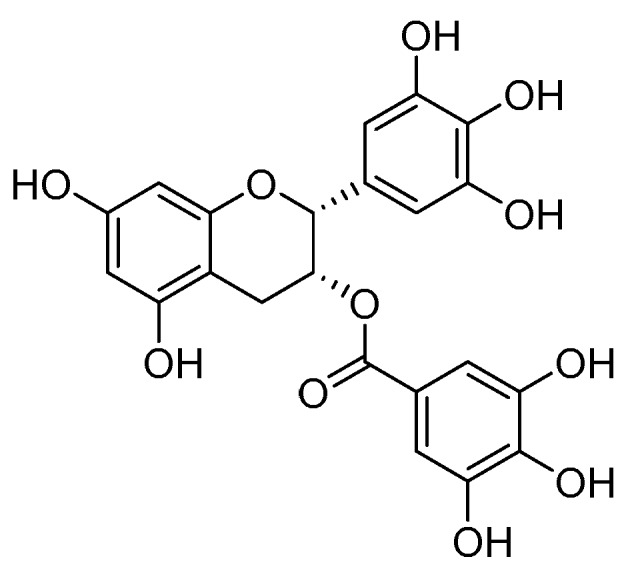
Chemical structure of epigallocatechin-3-gallate (EGCG).

Another commonly consumed beverage is tea (*Camellia sinensis* (L.) Kuntze). Infusions from tea are enormously rich in phenolic substances, and also contain considerable amounts of caffeine [199,200,201]. Consumption of tea was found to correlate with several health benefits including beneficial effects on the cardiovascular system [202]. Several studies showed that regular consumption of this polyphenol-rich beverage may exert cardio-protective effects in humans and reduce the risk of cardiovascular disease [203,204,205]. The phenolics of tea are represented particularly by epicatechin (EC), epigallocatechin (EGC), epicatechin-3-gallate (ECG), and epigallocatechin-3-gallate (EGCG) [206]. The effects of EGCG (Figure 3) are multifaceted and include among others the inhibition of the activator protein 1 (AP-1), the nuclear factor kappa B (NF-κB), the tumor necrosis factor α (TNFα) signaling, the inhibition of the vascular endothelial growth factor (VEGF) signaling, the insulin-like growth factor (IGF-1) signaling, and the activation of peroxisome proliferator-activated receptor (PPAR) [207].

The significance of dietary constituents in the context of metabolic and cardiovascular diseases is also evident in Table 2, which among herbal extracts also lists several prominent dietary constituents (e.g., curcumin and resveratrol).

Detailed studies on the efficacy of dietary constituents and the mechanisms by which they exert beneficial effects on cardiovascular and metabolic diseases are of critical importance in order to better rationalize dietary recommendations, and might also allow the development of novel effective nutraceuticals and functional foods [208,209,210,211,212]. Metabolism, bioavailability, and interaction with the intestinal microbiome will be important aspects to consider in this endeavor and also need to be taken into account for any natural product which is taken up orally.

## 7. Common Molecular Targets Affected by Natural Compounds in the Context of Cardiovascular and Metabolic Disorders

Diverse natural compounds have been shown to affect cardiovascular and metabolic disorders via different mechanisms, such as anti-inflammatory activity, improvement of blood lipid profiles, improvement of insulin sensitivity, or normalization of blood glucose levels [72,213,214,215,216,217]. Often the underlying molecular targets mediating these beneficial effects are not well understood. However, there are several molecular targets or pathways that are already well established to mediate the beneficial effects of natural compounds in the context of cardiovascular and metabolic disorders. Of those, selected examples, *i.e.*, the AMP-activated protein kinase (AMPK), cyclooxygenase (COX)-1 and -2, the dipeptidyl peptidase-4 (DPP-4), the endothelial nitric oxide synthase (eNOS), the transcription factors NF-κB, nuclear factor-erythroid 2-related factor 2 (Nrf2), and PPARγ, the protein-tyrosine phosphatase 1B (PTP1B), and 5-lipoxygenase (5-LO), are listed in Table 3, together with their major physiological consequences and some examples of compound classes of interacting natural products.

## 8. Natural Products (or Their Derivatives) Developed as Drugs for the Treatment of Cardiovascular and Metabolic Disorders

Other than providing a direct remedy, natural products also represent an excellent pool of inspiring lead structures for the development of successful pharmaceuticals to combat cardiovascular and metabolic disorders. This could be demonstrated with historical views on the development of the statins and the biguanides.

**Figure 4:**
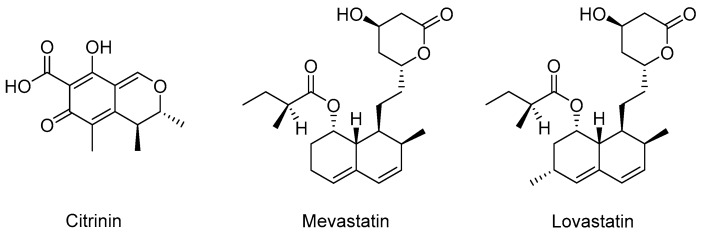
Chemical structures of natural inhibitors of cholesterol biosynthesis.

Aberrantly high cholesterol is causally connected to atherosclerosis and coronary heart disease. Therefore, in the 1950s and 1960s, companies were searching for compounds which block one of the 30 enzymatic reactions involved in cholesterol biosynthesis. However, none of the developed synthetic inhibitors of cholesterol biosynthesis had an ideal efficacy and safety profile [259]. In the early 1970s, the natural product citrinin (Figure 4) was isolated from fungi and identified as a potent inhibitor of 3-hydroxy-3-methylglutaryl coenzyme A (HMG-CoA) reductase [260], the rate-controlling enzyme in the cholesterol biosynthesis. Citrinin also displayed serum cholesterol lowering effects in rats [261]. Shortly after that, mevastatin (compactin; Figure 4), the first statin, was isolated from *Penicillium citrinum* [262]. It was found to be a strong HMG-CoA reductase inhibitor [263] with great structural similarity with HMG-CoA, the substrate of HMG-CoA reductase [259]. Mevastatin potently inhibited cholesterol biosynthesis *in vitro* and *in vivo* [262,264]. Clinical studies started in 1978 but it never came on the market due to side effects in dogs at a dosage of about 200 times the dosage used in human patients [259]. In the 1980s, clinical studies and long-term toxicity studies showed that lovastatin (Figure 4), a natural product isolated from *Aspergillus terreus* [265] and *Monascus ruber* [266], effectively lowered blood cholesterol levels and was well tolerated [259]. In 1987, lovastatin was approved by the FDA and became the first commercial statin. After lovastatin, several synthetic and semi-synthetic statins were also introduced to the market [259,267]. Today, statins represent the first-line pharmacologic intervention for dyslipidemia patients with failed treatment with diet and exercise alone [268] and are one of the most widely prescribed class of drugs worldwide.

**Figure 5:**
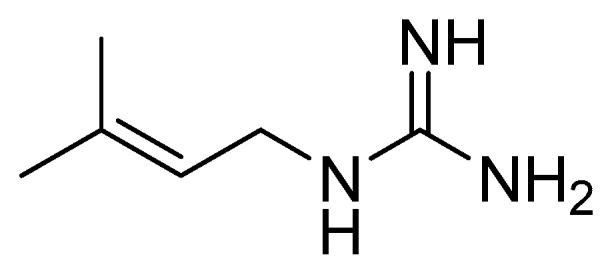
Chemical structure of the natural blood glucose lowering agent galegine.

Since the Middle Ages, *Galega officinalis* (also known as French lilac, Italian fitch, goat’s rue) has been known to relieve symptoms (the intense urination) of a disease now described as diabetes mellitus type 2 [98,269]. Galegine (Figure 5), a guanidine derivative which lowers blood glucose levels [270], turned out to be the bioactive constituent in *G. officinalis* [271,272]. Guanidine itself also decreases blood glucose levels [273], but is too toxic for clinical application. Galegine from *G. officinalis* is less toxic, nevertheless, clinical trials conducted with diabetic patients in the 1920s and 1930s, were not successful. However, the identification of the antidiabetic natural product galegine led to the development of the biguanide compound metformin, which is now one of the most important therapeutic agents for the treatment of diabetes mellitus type 2 [98,274,275].

## 9. Conclusions and Future Perspectives

The reviewed key examples and recent developments clearly demonstrate the great potential and the future promise of natural products for the treatment or prevention of cardiovascular and metabolic disorders. This work should provide an inspiration for authors who consider preparing further submissions to the special issue “Effects of Natural Products in the Context of Cardiometabolic Disease”. With the present review as well as with the expected valuable contributions to this special issue we do hope to further boost the scientific interest and knowledge on the efficacy of natural products with regard to the prevention and the therapy of cardiovascular and metabolic disease.

## Figures and Tables

**Table 1:** Medicinal plants targeting indications related to cardiovascular or metabolic disease.

Scientific Name of the Medicinal Plant	Common Name of the Medicinal Plant	Plant Organ	Indications
*Aesculus hippocastanum* L.	Horse-chestnut	Seeds	Venous insufficiency, varicose veins [21,22,23,24]
*Allium sativum* L.	Garlic	Bulbs/whole plant	Hypertension, hypercholesterolemia, diabetes mellitus type 2 [25,26,27,28,29,30]
*Aloe vera* (L.) Burm. f.	Aloe vera	Leaves	Diabetes mellitus type 2, hypercholesterolemia [31,32,33,34,35]
*Ammi visnaga* (L.) Lam.	Toothpick weed, bisnaga, khella	Fruits	Angina pectoris [36,37,38]
*Apocynum venetum* L.	Dogbane	Leaves	Hypertension [39,40,41,42]
*Artemisia dracunculus* L.	Tarragon	Leaves, aerial parts	Hyperglycemia [17,43,44,45]
*Artemisia herba-alba* Asso	White wormwood	Aerial parts	Hyperlipidemia, diabetes mellitus [46,47,48,49,50]
*Aspalathus linearis* (Burm. f.) R. Dahlgr.	Rooibos	Leaves	Diabetes mellitus type 2 [51,52,53,54]
*Astragalus membranaceus* Moench	Chinese milk vetch	Roots	Angina pectoris, atherosclerosis, diabetic nephropathy [39,55,56,57,58,59]
*Carthamus tinctorius* L.	Safflower	Flowers	Angina pectoris, hypertension, hyperlipidemia [39,60,61,62,63,64]
*Centaurium erythraea* Rafn	Common centaury	Whole plant, leaves	Diabetes mellitus [46,48,65,66,67]
*Cinnamomum cassia* (L.) J. Presl	Chinese cinnamon	Bark	Diabetes mellitus, diabetic nephropathy [68,69,70]
*Cinnamomum verum* J. Presl	Ceylon cinnamon	Bark	Diabetes mellitus type 2 [69,71,72,73,74]
*Commiphora mukul* (Hook. ex Stocks) Engl.	Gugal, guggul, gugul, Indian bdellium-tree, mukul myrrh tree	Resin	Hypercholesterolemia, hypertriglyceridemia [21,75,76]
*Coptis chinensis* Franch.	Chinese goldthread	Roots, flowers	Hypercholesterolemia, diabetes mellitus, non-alcoholic fatty liver disease [18,77,78,79]
*Coriandrum sativum* L.	Coriander	Seeds	Diabetes mellitus, hypercholesterolemia [80,81,82,83]
*Crataegus monogyna* Jacq./*C. oxyacantha* Jacq./*C. laevigata* (Poir.) DC./*C. pinnatifida* Bunge	Hawthorn	Sprigs with both leaves and flowers, fruits	Angina pectoris, atherosclerosis, hyperlipidemia [84,85,86,87]
*Cynara scolimus* L.	Globe artichoke	Leaves	Hypercholesterolemia [88,89]
*Fraxinus excelsior* L.	European ash	Fruits, seeds	Diabetes mellitus type 2, hepatic steatosis [90,91,92,93,94,95]
*Galega officinalis* L.	French lilac	Aerial parts	Diabetes mellitus [72,96,97,98]
*Gingko biloba* L.	Gingko, maidenhair tree	Leaves	Cerebrovascular disease, peripheral vascular disease, hypertension, diabetes nephropathy [75,85,99,100]
*Glycine max* (L.) Merr.	Soybean	Fruits, seeds	Diabetes mellitus, hyperlipidemia [101,102,103]
*Glycyrrhiza glabra* L.	Licorice	Roots	Atherosclerosis, hypercholesterolemia [85,104]
*Helianthus tuberosus* L.	Jerusalem artichoke	Tubers	Diabetes mellitus type 2, non-alcoholic fatty liver disease [105]
*Ilex paraguariensis* A. St.-Hil.	Yerba mate	Leaves	Obesity, diabetes mellitus [106,107,108,109,110,111]
*Lycium barbarum* L.	Chinese wolfberry	Fruits, roots	Diabetes mellitus, hyperlipidemia, hypertension [112,113,114,115,116,117,118,119]
*Momordica charantia* L.	Bitter melon	Fruits	Diabetes mellitus type 2 [109,120,121]
*Morus alba* L.	White mulberry tree	Root bark, leaves	Hyperglycemia [122,123,124,125,126,127]
*Nigella sativa* L.	Black cumin, black seed	Seeds, seed oil	Diabetes mellitus type 2, dyslipidemia [128,129,130,131,132]
*Ocimum sanctum* L.	Holy basil	Leaves, whole plant	Hypertension, dyslipidemia, diabetes mellitus [133,134]
*Olea europaea* L.	Olive	Leaves, fruit oil	Hypertension, atherosclerosis, diabetes mellitus, hepatic steatosis [135,136,137,138,139,140,141,142]
*Panax notoginseng* (Burkill) F.H. Chen ex C.H. Chow	Notoginseng, pseudoginseng	Roots	Angina pectoris, coronary artery disease [21,75,143]
*Rauvolfia serpentina* (L.) Benth. ex Kurz	Indian snakeroot	Roots	Hypertension [75,144,145]
*Rhodiola rosea* L.	Golden root	Roots	Angina pectoris, ischemic heart disease [39,146,147]
*Rosmarinus officinalis* L.	Rosemary	Leaves	Capillary permeability and fragility disturbances [21,148,149]
*Ruscus aculeatus* L.	Butcher’s broom	Rhizomes	Venous insufficiency, varicose veins [21,150]
*Sambucus nigra* L.	European elder, black elder	Flowers	Diabetes mellitus type 2 [151,152,153]
*Schisandra chinensis* (Turcz.) Baill.	Five-flavor berry	Fruits, seeds	Hypertension, myocardial infarction, hyperlipidemia, diabetic nephropathy, diabetes mellitus [154,155,156,157,158]
*Silybum marianum* (L.) Gaertn.	Milk thistle	Seeds, aerial parts	Diabetes mellitus type 1 and 2 [90,159,160,161,162,163]
*Stevia rebaudiana* (Bertoni) Bertoni	Sweet leaf, candyleaf	Leaves	Diabetes mellitus type 2 [164,165,166,167]
*Trigonella foenum-graecum* L.	Fenugreek	Seeds	Metabolic syndrome, diabetes mellitus type 2 [168,169]
*Vaccinium* spp.	Blueberries	Fruits, leaves	Diabetes mellitus type 2, metabolic syndrome [96,109,170,171,172,173]
*Veratrum album* L./*V. nigrum* L./*V. japonicum* (Baker) Loes./*V. viride* Aiton	False helleborine/black false hellebore	Rhizomes	Hypertension [75,174,175]
*Viscum album* L.	Mistletoe	Aerial parts	Hypertension [176]

**Table 2:** Herbal extracts and natural products in recruiting clinical trials targeting indications related to metabolic or cardiovascular diseases ^1^.

Name of the Product	National Clinical Trial (NCT) Identifier	Phase	Studied Condition
BeneFlax^®^ (Flaxseed (*Linum usitatissimum* L.) lignans)	NCT02391779	Phase 2	Hypertension
Biscuit containing “Kothala Himbutu” (*Salacia reticulata* Wight)	NCT02290925	Phase 3	Diabetes mellitus type 2
*Coleus forskohlii* (Willd.) Briq.	NCT02143349	Phase 3	Risk factors of metabolic syndrome
Combined Rg3-enriched Korean red ginseng and American ginseng	NCT01578837	Phase 1 and 2	Diabetes mellitus type 2, hypertension
Curcumin	NCT01968564	- ^2^	Vascular aging
Curcumin	NCT02529982	Phase 2	Non insulin dependent diabetes
Curcumin	NCT02529969	Phase 2	Non insulin dependent diabetes
Dantonic^®^ (T89)	NCT01659580	Phase 3	Angina pectoris
Euiiyin-tang	NCT01724099	Phase 2 and 3	Obesity
Fibre grain herb	NCT02553382	Phase 3	Diabetes mellitus type 2
“Fu-zheng-qu-zhuo” oral liquid	NCT02044835	Phase 2 and 3	Ischemic nephropathy
Ginger	NCT02289235	Phase 0	Non-alcoholic fatty liver disease
*Phyllanthus niruri* L. and *Sida cordifolia* L. (Vedicine)	NCT02107469	-	Diabetic peripheral polyneuropathy
Quercetin	NCT00065676	Phase 2	Diabetes mellitus, obesity
Red grapes polyphenol supplementation	NCT02633150	-	Obesity, insulin resistance
Resveratrol	NCT02245932	Phase 3	Overweight
Resveratrol	NCT01564381	Phase 1 and 2	Cardiovascular disease
Resveratrol	NCT01842399	Phase 1 and 2	Vascular resistance, hypertension
Resveratrol	NCT02246660	-	Peripheral arterial disease
Resveratrol	NCT02137421	-	Metabolic syndrome, coronary artery disease
Resveratrol	NCT02129595	-	Pre-diabetes
Resveratrol	NCT01997762	Phase 4	Gestational diabetes
Resveratrol	NCT02216552	Phase 2 and 3	Non-alcoholic fatty liver disease, diabetes mellitus type 2, metabolic syndrome
Resveratrol	NCT02419092	-	Obesity
Resveratrol	NCT01881347	-	Diabetes mellitus
Resveratrol	NCT02549924	Phase 2	Diabetes mellitus type 2
Resveratrol	NCT02244879	Phase 3	Diabetes mellitus type 2, inflammation, insulin resistance

^1^ Information retrieved from www.clinicaltrials.gov on 21 January 2016; ^2^ “-“ indicates that there is no information for the phase provided on the corresponding trial page at www.clinicaltrials.gov.

**Table 3:** Selected molecular targets relevant for cardiovascular and metabolic disorders, which are well known to be affected by diverse natural products.

Molecular Target/Pathway	Major Physiological Consequence	Selected Compound Classes of Interacting Natural Products
AMPK	Activation leads among others to inhibition of fat and cholesterol synthesis, promotion of fat oxidation, enhancement of mitochondrial biogenesis, and promotion of glucose uptake in skeletal muscle and fat cells	Alkaloids, chalcones, flavonoids and other polyphenols, galegine, salicylate, terpenoids [214,218,219,220,221]
COX-1/-2	Inhibition leads to reduced biosynthesis of pro-inflammatory prostaglandins	Alkaloids, stilbenes, flavonoids and other polyphenols, terpenoids [222,223,224]
DPP-4	Inhibition leads to decreased incretin degradation (and thus increased insulin secretion)	Alkaloids, flavonoids and other polyphenols, polypeptides, terpenoids [225,226,227]
eNOS	Activation leads to increased availability of anti-inflammatory nitric oxide (NO), a major antiatherogenic factor in the vasculature	Anthocyanidins, fatty acids, flavonoids and other polyphenols, ginsenosides, triterpenoic acids [228,229,230,231,232,233,234]
NF-κB pathway	Inhibition leads to impaired expression of pro-inflammatory mediators	Alkaloids, curcuminoids, chalcones, diterpenes, flavonoids, iridoids, naphtoquinones, salicylates, sesquiterpene lactones, stilbenes, triterpenes [235,236,237,238,239]
Nrf2 pathway	Activation leads to increased expression of cytoprotective (e.g., antioxidant) and reduced expression of lipo-and gluconeogenic genes	Carotenoids, chalcones, curcuminoids, diterpenes, flavonoids and other polyphenols, isothiocyanates, phytoprostanes, sesquiterpenes, sesquiterpene lactones, triterpenes [240,241,242,243]
PPARγ	Activation leads to insulin sensitization and normalization of blood glucose levels	Amorfrutins, diterpenequinones, flavonoids, neolignans, polyacetylenes, sesquiterpene lactones, stilbenes [244,245,246,247,248,249]
PTP1B	Inhibition leads to prolonged and enhanced insulin and leptin signaling (increased insulin sensitivity and reduced food intake)	Alkaloids, bromophenols, chalcones, coumarins, diterpenes, flavonoids, lignans, *N*- or *S*-containing compounds, sesquiterpenes, sesterterpenes, steroids, triterpenes [250,251,252,253,254]
5-LO	Inhibition leads to reduced biosynthesis of pro-inflammatory leukotrienes	Alkaloids, coumarins, depsides, quinones, flavonoids and other polyphenols, polyacetylenes, sesquiterpenes, triterpenes [222,255,256,257,258]

## Abbreviations

The following abbreviations are used in this manuscript:

5-LO	5-Lipoxygenase
AMPK	AMP-Activated Protein Kinase
AP-1	Activator Protein 1
COX-1/2	Cyclooxygenase-1/2
DPP-4	Dipeptidyl Peptidase-4
eNOS	Endothelial Nitric Oxide Synthase
FDA	US Food and Drug Administration
HMG-CoA	3-Hydroxy-3-Methylglutaryl Coenzyme A
HTS	High Throughput Screening
IGF-1	Insulin-Like Growth Factor
LDL	Low-Density Lipoprotein
NCT	National Clinical Trial
NF-κB	Nuclear Factor Kappa B
Nrf2	Nuclear Factor-Erythroid 2-Related Factor 2
NO	Nitric Oxide
PPAR	Peroxisome Proliferator-Activated Receptor
PTP1B	Protein-Tyrosine Phosphatase 1B
TNFα	Tumor Necrosis Factor α
VEGF	Vascular Endothelial Growth Factor
WHO	World Health Organization
